# Implementation of Medicines Pricing Policies in Ghana: The Interplay of Policy Content, Actors’ Participation, and Context

**DOI:** 10.34172/ijhpm.2023.7994

**Published:** 2023-10-11

**Authors:** Augustina Koduah, Leonard Baatiema, Irene A. Kretchy, Irene Akua Agyepong, Anthony Danso-Appiah, Anna Cronin de Chavez, Timothy Ensor, Tolib Mirzoev

**Affiliations:** ^1^Department of Pharmacy Practice and Clinical Pharmacy, School of Pharmacy, University of Ghana, Legon, Ghana.; ^2^Department of Health Policy, Planning & Management, School of Public Health, University of Ghana, Legon, Ghana.; ^3^Public Health Faculty, Ghana College of Physicians and Surgeons, Accra, Ghana.; ^4^Department of Epidemiology and Disease Control, School of Public Health, University of Ghana, Legon, Ghana.; ^5^London School of Hygiene and Tropical Medicine, London, UK.; ^6^Nuffield Centre for International Health, University of Leeds, Leeds, UK.; ^7^Department of Global Health and Development, London School of Hygiene and Tropical Medicine, London, UK.

**Keywords:** Access to Medicines, Ghana, Medicines Pricing Policy, Medicines Price Control, Policy Implementation

## Abstract

**Background:** Implementing medicines pricing policy effectively is important for ensuring equitable access to essential medicines and ultimately achieving universal health coverage. However, published analyses of policy implementations are scarce from low- and middleincome countries. This paper contributes to bridging this knowledge gap by reporting analysis of implementation of two medicines pricing policies in Ghana: value-added tax (VAT) exemptions and framework contracting (FC) for selected medicines. We analysed implications of actor involvements, contexts, and contents on the implementation of these policies, and the interplay between these. This paper should be of interest, and relevance, to policy designers, implementers, the private sector and policy analysts.

**Methods:** Data were collected through document reviews (n=18), in-depth interviews (n=30), focus groups (n=2) and consultative meetings (n=6) with purposefully identified policy actors. Data were analysed thematically, guided by the four components of the health policy triangle framework.

**Results:** The nature and complexity of policy contents determined duration and degree of formality of implementation processes. For instance, in the FC policy, negotiating medicines prices and standardizing the tendering processes lengthened implementation. Highly varied stakeholder participation created avenues for decision-making and promoted inclusiveness, but also raised the need to manage different agendas and interests. Key contextual enablers and constraints to implementation included high political support and currency depreciation, respectively. The interrelatedness of policy content, actors, and context influenced the timeliness of policy implementations and achievement of intended outcomes, and suggest five attributes of effective policy implementation: (1) policy nature and complexity, (2) inclusiveness, (3) organizational feasibility, (4) economic feasibility, and (5) political will and leadership.

**Conclusion:** Varied contextual factors, active participation of stakeholders, nature, and complexity of policy content, and structures have all influenced the implementation of medicines pricing policies in Ghana.

## Background

**Key Messages**

**Implications for policy makers**

- Having clearly defined medicines pricing policy objectives is equally important for effective implementation, as having legal and organizational structures to implement these objectives.
- A clear legitimacy and mandate to regulate medicines pricing are critical for implementation, but there is also a need to balance multiple agendas and priorities of different policy actors.
- Appropriate sequencing of tasks to be performed by stakeholders during medicines pricing policy implementation can be salient and demands attention, especially in participatory decision-making.
- A clear and structured process can be a useful facilitator of policy implementation but needs to be simple enough for the policy actors to easily navigate the processes.

**Implications for the public**

Availability of quality, safe and affordable medicines is vital for achievement of universal health coverage. National policies are often designed and implemented by governments to reduce medicines prices and make quality and safe medicines available to all. The government of Ghana introduced two such policies: value-added tax (VAT) exemptions for selected medicines and framework contracting (FC) for high-demand medicines. Initially, some medicine prices were reduced as planned, but numerous challenges such as the depreciation of the Ghanaian currency, unpredictable pharmaceutical supply chain system due to the COVID-19 pandemic, and indebtedness of some health facilities, have resulted in delays in medicines supply. Detailed analyses of implementation processes, such as the one reported in this article, can help improve implementation processes, and ultimately contribute to improved access to essential medicines.

Ensuring universal and equitable access to essential medicines is an important contributor towards universal health coverage in low- and middle-income countries (LMICs). Successful implementation of effective health policies is a critical mechanism by which national governments can address their population’s health needs, especially the poorest and most vulnerable.^1,2^

 Medicine pricing policies are priorities of most governments in LMICs,^3-5^ and can target various stages of the supply chain, from manufacturing to retailing.^1,6,7^ The use of a regulatory framework is a major approach to managing medicines prices.^8-12^ For example, at the manufacturing end, pharmaceutical companies can be incentivized by the government through policies such as value-added tax (VAT) exemptions or bulk purchases to reduce their ex-factory prices.^1,3^ At the retail level, prices can be regulated through standardized markup regimes and the promotion of substitution of high-priced branded medicines with quality-assured generic and biosimilar medicines.^3,8^ Additionally, governments can regulate the entry of high-priced medicines through cost or risk sharing.^13^

 Implementations of medicines pricing policies typically involve diverse policy actors including government,^14-16^ wholesalers,^10,17^ manufacturers,^15-18^ professional bodies^11,16,19^ public and private health facilities.^11,20^ Actors’ engagements in policy implementation are influenced by multiple contextual enablers or barriers such as the presence (or absence) of robust implementation and impact monitoring systems^21,22^ and the related (in)adequate policy-makers’ understanding of expected outcomes.^10,23^ Understanding the context can therefore help answer important questions related to how and why the implementation of health policies occurs and what understanding it can shed on the observed versus the desired policy outcomes.

 Policy implementation is complex and the processes, actors, context, and content of policy interact to influence achievement of policy outcomes.^24^ An in-depth understanding of medicines pricing policies, including their implementation processes, actors involved, and contextual influences can therefore inform interventions to improve effectiveness of these policies in improving access to medicines. While research exists on the implementation of medicine pricing policies covering processes,^8,10,11^ actors’ involvement,^12,16,19^ and contextual barriers and enablers,^20,25,26^ this body of knowledge remains rather fragmented and comprehensive analyses are particularly limited from LMICs.

 Since 2017, the Government of Ghana has tried to improve medicine pricing and access to essential medicines (ie, medicines that satisfy healthcare needs of Ghanaians)^27^ by implementing policies related to VAT exemptions for selected imported pharmaceutical products and a framework contracting (FC).^28,29^ The expected outcome of the VAT exemption policy is a 30% reduction in the National Health Insurance Scheme (NHIS) medicines tariffs,^30-32^ whereas the intended outcome of the FC policy is a centralized procurement process for bulk purchase and negotiated reduced prices of high-demand essential medicines.^33^

 The VAT exemption policy involved the review of the 2015 VAT Exemption Regulations (L.I 2218),^34^ amendment of the VAT law,^35^ and promulgation of the VAT Exemption Regulations 2017 (L.I 2255).^28^ A total of 552 Active Pharmaceutical Ingredients (APIs,) manufacturing inputs and packaging materials, and 483 imported finished pharmaceutical products were exempted from VAT.^28^ Under the FC policy, an agreement was made with selected vendors to supply essential medicines at negotiated prices.^33^ The FC policy was a recommendation of the 2012 Health Commodity Supply Chain Master Plan.^36^ Both VAT exemptions and FC policies have been implemented since 2018, and therefore represent relevant case studies to understand the implementation of medicines pricing policies in Ghana.

 This paper seeks to contribute to bridging the aforementioned knowledge gaps by reporting our analysis of the implementation of VAT exemptions for selected imported pharmaceutical products and the FC in Ghana. Our research questions were: In what way did the policy contents for VAT exemptions and FC policies shaped the implementation (specifically, duration and degree of formality) of these policies? How has involvement of policy actors shaped the implementation of medicines pricing policies? Which contextual enablers and barriers affected the implementation of these policies? In what way has the interplay between contents, context and actors affected the implementation of medicines pricing policies and the achievement of intended policy outcomes?

 We hope this paper will be of interest, and relevance, to different stakeholders including designers and implementers of medicines pricing policies, private sector stakeholders interested in engaging with the implementation of national or sub-national policies, and scholars interested in advancing their understanding of policy analysis in LMICs.

## Methods

 This paper builds on and consolidates, results of our systematic review^5^ and stakeholder analyses^16^ from an AMIPS (Access to Medicines through Improved Pricing Strategies) study which examined implementation of four medicines pricing policies in order to inform improved policy implementation and contribute to improved access to essential medicines in Ghana. A cross-sectional qualitative study design was used.

###  Data Collection

 Data were collected through document reviews, in-depth interviews (IDIs), focus group discussions (FGDs), and consultative meetings with purposefully identified informants.

 Using policy document reviews, we mapped the contents of each policy, their implementation processes, policy actors involved and revealed documented barriers and enablers. The documents were sourced from the Ministry of Health (MoH) website, MoH Pharmacy Directorate, and Google Scholar. We reviewed legislative instruments, Parliament reports, NHIS Medicines List, and technical working group (TWG) meeting reports (n = 18). Our main inclusion criterion was relevance to FC and VAT exemptions for pharmaceutical products. Documents with core focus on other medicines’ pricing policies were excluded. Examples of specific documents included the report of the Committee on Subsidiary Legislation on the VAT Exemptions Regulations (L.I 2255) (October 2017), the Local Pharmaceutical Production Committee on the review of restricted list report (December 2016), and procurement of essential medicines through FC report (November 2019).

 A total of 30 IDIs were conducted from August 2020 to March 2021, to understand FC and VAT exemptions policy implementation processes, actors involved, and contextual influences. The anonymized respondents’ profiles are shown in Table 1.^16^ Respondents were purposefully identified from the documents and using snowballing from the previous IDIs and consultative meetings. A question guide was utilised for the IDIs (See Supplementary file 1), which was semi-structured by implementation processes, timelines, actors involved, and contextual barriers and enablers to policy implementation. The IDIs were conducted via telephone, zoom, or in person as feasible, and each preceded by obtaining verbal or written informed consent. All IDIs were in English, lasted on average 45 minutes, were digitally recorded, transcribed verbatim, and anonymised for analysis.

**Table 1:** List of Sectors and Agencies of Respondents^16^

**Sector **	**Institution **	**Respondents **
Government agencies	GRA	1
	MoH	5
	MoF	1
	NHIA	1
	GHS-RHD	1
	GHS-RMS	1
	GHS-HQ	2
Service providers	Teaching hospital	1
	Regional health facility	1
	Public hospital	3
	Public polyclinic	1
	Private hospital	1
	Christian Health Association of Ghana	1
Development partner	WHO	1
Professional association	PSGH	1
	Society of Private Medical and Dental Practitioners	2
Pharmaceutical industry	Pharmaceutical Manufacturers Association of Ghana	2
	CPPA	1
	Pharmaceutical wholesaler/importer/retailer	1
NGO	Coalition of Non-Governmental Organizations in Health	1
	Ghana NCDs Alliance	1

Abbreviations: GRA, Ghana Revenue Authority; MoH, Ministry of Health; MoF, Ministry of Finance; NHIA, National Health Insurance Authority; GHS-RHD, Ghana Health Service Regional Health Directorate; GHS-RMS, Ghana Health Service Regional Medical Store; GHS-HQ, Ghana Health Service Headquarters; WHO, World Health Organization; PSGH, Pharmaceutical Society of Ghana; CPPA, Community Pharmacy Practice Association; NCDs, Noncommunicable diseases; NGO, non-governmental organization.

 Two urban health facilities were purposively selected because we were able to obtain the requisite numbers of participants for an FGD and the required space to observe all COVID-19 safety protocols as prescribed by the Ghana Health Service (GHS). The FGDs were conducted in February 2021 with public healthcare professionals to understand their views on the policy implementation processes, barriers, and enablers. The FGDs were held in Accra, and in each facility, ten individuals with professional backgrounds in procurement and supplies, purchasing, administration, and pharmacy participated. They were conducted in person, lasted on average 1 hour and 20 minutes, and consent was obtained for participation and to record proceedings. The discussion notes were transcribed verbatim and anonymised for analysis.

 Six consultative meetings were held throughout the study to communicate and validate emerging findings with relevant stakeholders. The meetings were held between October 2020 and May 2021 and involved the National Medicines Pricing Committee (NMPC) (October 2020, April 2021), pharmaceutical sector stakeholders (December 2020), medicine price mark-up working group (December 2020), Society of Private Medical and Dental Practitioners (February 2021) and NMPC and pharmaceutical sector stakeholders (May 25, 2021). These meetings were organised in collaboration with the MoH Pharmacy Directorate. The MoH invited attendees and meetings lasted four hours on average. During meetings, the MoH representatives and members of the study team led discussions on medicines pricing implementation processes, barriers and enablers, potential solutions, and the uptake of study findings for policy and practice. The researchers took notes at all meetings to reduce recall bias, which were subsequently analysed alongside formal minutes. To further address potential recall bias and improve quality, data sourced from interviews, document reviews, and consultative meetings were triangulated.

###  Data Analysis

 Thematic content analysis was used, which followed a framework method for analyzing qualitative data.^37^ Framework method involves seven steps: (1) transcription, (2) familiarization with the data, (3) coding, (4) developing a working analytical framework, (5) applying the analytical framework, (6) charting data into the framework matrix, and (7) interpreting the data.

 Following transcription, the anonymized interview transcripts, documents reviews, and researchers’ notes from the consultative meetings were initially examined by AK and LB to familiarize with the whole dataset. The data was then coded for analysis using the four components of the Walt and Gilson’s health policy triangle: actors, contents, contexts, and processes.^38^

 The policy triangle constituted our working analytical framework, to help explain how and why the policies were implemented, and key influences on policy processes from actors, contents, and contexts. Actors denote individuals, organizations, groups and governments and their actions and inactions that affect the policy. Content refers to the substance of the policy which details its constituent part. Context includes systemic factors such as political, economic, social or cultural, national, and international which may have influenced the policy implementation. Processes refer to ways in which a policy is initiated, developed, and implemented.^38^ The four parameters of the policy triangle are interrelated, for example, actors’ (in)actions are influenced by the context in which policies are implemented. Policy actors can exercise their power to influence policy decisions as they engage in the policy process^16^ and can sometimes form alliances through negotiation, consultation, and consensus building. ^39^ The policy content can shape implementation modalities, for example through building mechanisms for rapport and trust with communities. Contextual environment influences implementation processes, contents, and actors.^38^ To better understand the context, we relied on the three-tier framework comprising macro (national and international political and economic influences), meso (organizational practices and structures), and micro (individual interests and preferences) levels.^40^

 The analytical framework was then applied to the coded interview transcripts, insights from documents reviews, and researchers’ notes from the consultative meetings, to identify common patterns of meaning based on the research questions. The emerging results were further grouped and reviewed to generate a thematic map of the findings, corresponding to the four themes and reflecting our research questions ie, (1) policy content influence on VAT exemption and FC policy implementation processes, (2) actors roles/influence on VAT exemption and FC policies implementation processes, (3) contextual factors influence on VAT exemption and FC policies implementation processes, and (4) interplay between policy content, actors’ roles and context on policy implementation outcomes.

 The findings were further interpreted, specifically examining potential linkages between the four themes to identify the interplay between policy contents, actors and contexts in relation to the policy implementation and achievement of policy outcomes.

## Results

 The four analytical themes relate to our research questions and provide a structure for our results. Following reporting influences of contents, actors and then contexts on policy implementation, we reflect on the complex interplay between these in shaping the implementation processes.

Table 2 summarizes the influences of policy contents, actors, and context on the implementation processes and resulting policy outcome for the FC and VAT exemption policies. Each of these is further explained in the next sections.

**Table 2:** Summary of Influence of Policy Content, Policy Actors and Context on Implementation Process and the Resulting Policy Outcomes

**Policy and** **Expected Outcome**	**Implementation Year**	**Content Influence on Implementation Processes**	**Actors Influences on Implementation processes**	**Contextual Influences on Implementation Processes**		**Resulting Outcomes**
				**Enablers**	**Constraints**	
FC/Centralised procurement process for high demand essential medicines and negotiated lower prices	2018	The nature and complexity of policy content ie, to create centralised procurement for selected and negotiate lower prices for high medicines influenced the length and duration of the implementation process as shown in Figure 1. This lasts on average a year	Involvement of several actors influenced the degree of participation and efficiency. For example, 1. RHAs, RMSs, THs, NHIA, FDA, GHSC-PSM, MoH, P&SC, MoH-Pharmacy, and GHS-SSDM (ie, TWG) – selected the high volume, high value, and high supply risk essential medicines for centralised procurement and price negotiation – determined required quantities – prepared tender documents – publically received tenders – determined the capacity of vendors to supply selected medicines – negotiated lower prices. See Figure 3 for pricing models 2. Minister of Health, THs Chief Executive Officers and GHS Director General and selected vendors – signed MOU 3. RHAs and THs – signed contracts with the selected vendors to supply the selected essential medicines 4. Vibrant local pharma industry – participated in national competitive tendering processes	1. Political support – the Minister of Health facilitated the implementation process by signing an MOU 2. Institutional capacity of the MoH and agencies to negotiate prices – the collaborative approach toward FC implementation was a facilitator 3. Administrative capacity of health facilities – some facilities ring-fenced drug revolving fund for FC payment 4. Other policies – eg, public procurement laws	1.Economic factors – depreciation of local currency – increasing inflation rate – high bank interest rate 2. High degree of outlier prices quoted by the pharmaceutical industry during the tendering process – organizational interest to make more profits – some vendors did not supply medicines at the agreed prices 3. Administrative (in)capacity of health facilities – high indebtedness of some facilities – unable to call-off all the quantities agreed on 4. COVID-19 pandemic – delayed global supply chain	54 Essential medicines lower prices negotiated and centrally procured under framework with 8% upward price adjustment
	2019					65 Essential medicines lower prices negotiated and centrally procured under framework with 20% upward price adjustment
	2020-2021					65 Essential medicines lower prices negotiated and centrally procured under framework
VAT exemption/At least 30% reduction in NHIS listed medicines prices	2018	The degree of formality and straight- forwardness of the Parliament approved policy content and aim ie, remove VAT from 552 APIs, manufacturing inputs and packaging materials and 483 imported finished pharmaceutical products shaped the length and duration of the implementation process as shown in Figure 2. This lasts on average two weeks	Multisectoral organizational involvement beyond the health sector influences the degree of participation and efficiency. For example, 1. MoH, GNCOP, PSGH, MOTI, NHIA, PMAG, FDA, WHO, GRA, CPPA, GHS (ie, TWG) – selected pharmaceutical products for VAT exemption – coordinated implementation processes 2. Parliament of Ghana – approved the Legislative Instrument L.I 2255 for implementation 3. GRA an agency of the MoF – hosts the ICUMS online portal (https://gra.gov.gh/customs/icums/) – approves VAT exemption application 4. Vibrant local pharmaceutical industry – importation of API, manufacturing inputs and finished products	1. High political support – VAT exemption for selected pharmaceutical stated in the 2016 Manifesto of the government in power, 2017 national budget statement – the Minister of Health facilitated implementation process 2. Other policies facilitated timely implementation – VAT regulations 2015 (L.I. 2218) – Public Procurement Laws 3. Institutional capacity of GRA – VAT exemption applied for on the ICUMS online portal 4. Collaborative approach to policy implementation – multi-sectoral	1. Economic factors – depreciation of local currency – increasing inflation rate – high bank interest rate 2. Institutional process – Pharmaceutical companies apply individually for exempted products on the ICUMS portal and this time-consuming especially if one imports several products	533 NHIS 2018 listed medicines prices reduced by at least 30%. 11 NHIS 2018 listed medicines prices reduced by more than 30%. 66 NHIS 2018 listed medicines had higher prices than expected

Abbreviations: API, active pharmaceutical ingredients; CPPA, Community Pharmacy Practice Association; FDA, Food and Drugs Authority; FC, framework contracting; GHS, Ghana Health Service; GHS-SSDM, Ghana Health Service Supplies Stores and Drug Management; GNCOP, Ghana National Chamber of Pharmacy; GRA, Ghana Revenue Authority; GHSC-PSM, Global Health Supply Chain-Procurement and Supply Management; ICUMS, Integrated Customs Management Systems; MoF, Ministry of Finance; MoH, Ministry of Health; NHIA, National Health Insurance Authority; NHIS, National Health Insurance Scheme; PMAG, Pharmaceutical Manufacturers Association of Ghana; PSGH, Pharmaceutical society of Ghana; MOTI, Ministry of Trade and Industrial; TWG, Technical Working Group; P&SC, Procurement and Supply Chain; RHAs, Regional Health Administrations; RMSs, Regional Medical Stores; THs, teaching hospitals; VAT, value-added tax; WHO, World Health Organization; MOU, memorandum of understanding.

###  Influences of Policy Contents on Implementation Processes

**Figure 1:**
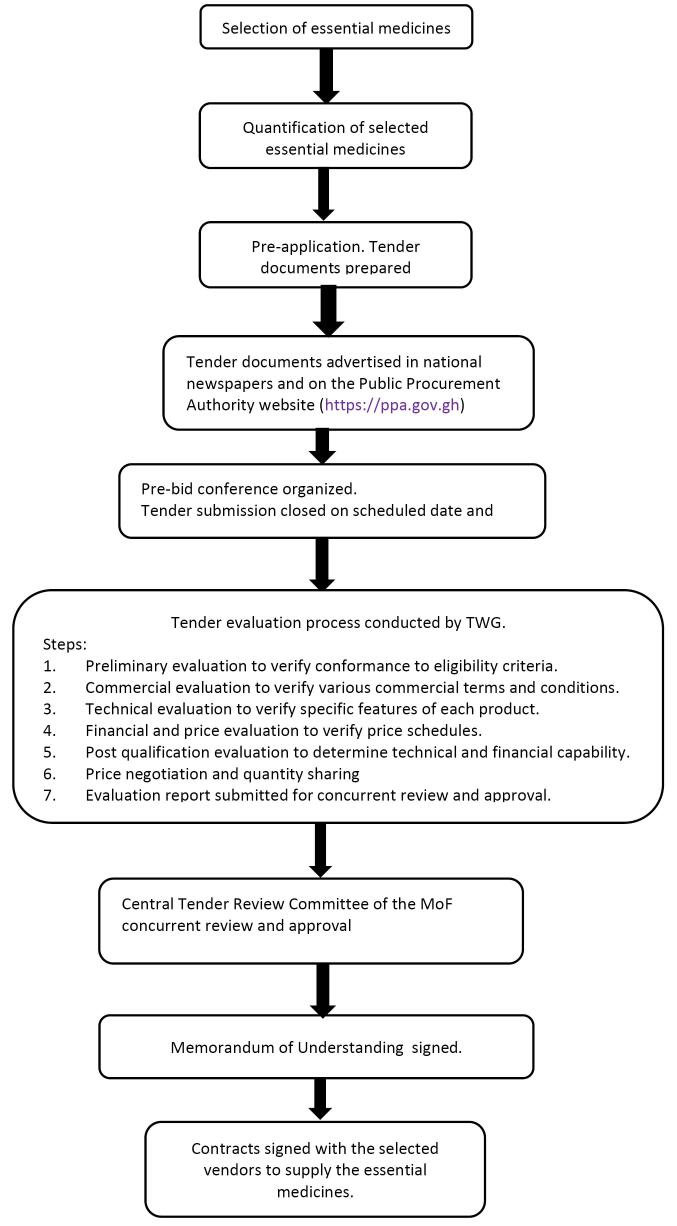
Schematic Outline of Framework Contracting Implementation Process. Abbreviations: MoF, Ministry of Finance; TWG, Technical Working Group.

**Figure 2:**
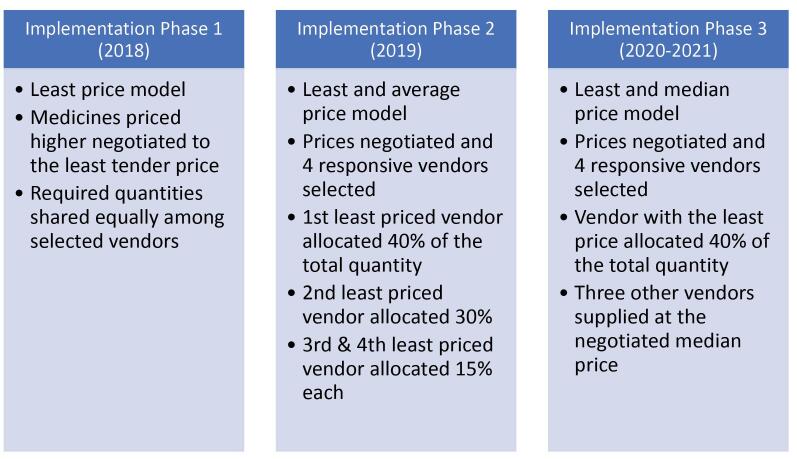
Framework Contracting Implementation Pricing Model.

**Figure 3:**
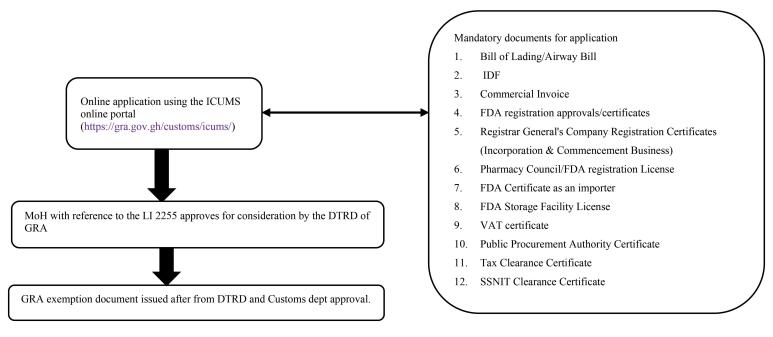
Schematic Outline of Value-Added Tax Exemptions Application Process. Abbreviations: FDA, Food and Drugs Authority; GRA, Ghana Revenue Authority; ICUMS, Integrated Customs Management Systems; MoH, Ministry of Health; VAT, value-added tax; DTRD, Domestic Tax Revenue Division; IDF, Import Declaration Form; SSNIT, Social Security and National Insurance Trust.

 Our analysis revealed two main implications of how policy contents (ie, nature and complexity) affected policy implementations: effects on implementation duration and degree of formality of implementation processes. The FC policy — which sought to create a centralized procurement process for the bulk purchase of selected essential medicines and negotiate medicine prices with vendors and by so doing standardize the tendering processes for the framework agreement — had a long implementation duration. The complex nature of the FC policy shaped the implementation structures and demanded a long process of selection, quantification, tendering evaluation, and negotiation of pricing model with several vendors per each implementation cycle (Figures 1 and 2). The FC implementation has gone through three phases with high volume, high value and high supply risk essential medicines selected and quantified by key policy actors (See Supplementary file 2 for FC selected essential medicines). On the other hand, VAT exemption policy implementation required a much simpler process, involving an application for VAT exemptions with the required documentation as shown in Figure 3. Data from interviews showed that responsible agencies in a sequential manner reviewed and approved applications and if all required documents were accurate and uploaded, approval was obtained within an average of 14 days.

 The policy content also influenced the degree of formality of the implementation processes. The VAT exemption policy is Legislative Instrument 2255, a revised version of the Legislative Instrument 2218 approved by the Parliament of Ghana for implementation. Because LI 2255 is a revised version, there were already existing implementation structures to support the process, as noted by one respondent,

 “*There were existing structures put in place for Ghana Revenue Authority to provide VAT exemptions to qualified applicants*” [Public Sector].

 During FC policy implementation there were formal engagements with stakeholders as mandated by the Public Procurement Authority standard tender documents, however, the required meetings with vendors rather lengthened the implementation.

###  Influences of Policy Actors on the Implementation Processes

 The main influence of policy actors over policy implementation is degree of actors’ participation and resultant implications on efficiency of decision-making. There was a high degree of participation of varied stakeholders for FC and VAT exemption policies, creating avenues for decision-making and promoting inclusiveness. TWGs with multiple stakeholders were created for both policy implementation processes (see Table 2) and these TWGs made decisions on selected medicines for implementation, tendering processes, exemptions applications, price negotiations, and coordination of the implementation processes.

 “*We tried to involve all relevant stakeholders from the beginning, and this was very important for the Ministry of Health to have engaging and participatory processes with all hands-on deck”* [Public Sector].

 As national policies, there was inherent multi and cross-sectoral collaboration, however, the VAT exemption policy implementation was more cross-sectoral because of its nature and therefore involved a wider range of actors such as the Parliament which provided high political support. On the other hand, the FC policy was more ‘technical’ with several steps and decisions around tendering, procurement, and price negotiations requiring actors with technical expertise in tendering, procurement, and price negotiations.

 Although implementation processes were generally dominated by government agencies, as some of these agencies are mandated to ensure access to health and/or facilitate the implementation, notably non-state actors such as pharmaceutical industry were involved in the implementation decisions. The local pharmaceutical industry actors participated in the national competitive tendering process and importation of APIs, manufacturing inputs, and finished products. Further details of the policy actors’ powers, engagement, and resultant influences over FC and VAT exemptions are reported elsewhere.^16^

###  Contextual Influences on the Implementation Process

 Three contextual enablers were identified from our analysis. First, high political support and will. For instance, the minister of health signed the memorandum of understanding (MOU) with the vendors as his commitment to the FC implementation. Also, the intent to abolish the 17.5% VAT on selected finished imported medicines not produced in Ghana was stated in the 2016 manifesto of the government in power.^41^ This intention was further captured in the national 2017 Budget Statement and Financial policy with the Ministries of Finance and Health tasked to implement.^30^

 “*There was high political will and increased leadership commitment for VAT exemption policy implementation and the Parliament was supportive” *[Professional Association].

 Second, the implementation of already existing policies. The existence of VAT Regulations 2015 (L.I 2218)^34^ allowed for timely promulgation of the VAT (Amendment) Regulation 2017 (L.I 2255)^28^ which spelled out the selected pharmaceutical products, raw materials, and packaging materials to be exempted. Additionally, existing national structures, for example, the Public Procurement Laws (Act 663 and Act 914)^42,43^ and national medicines policy^4^ augmented the FC policy implementation in terms of promoting access to quality affordable medicines through a national competitive tendering process.

 Third, institutional capacities. The sufficient capacity of the MoH together with its agencies such as the National Health Insurance Authority (NHIA) to negotiate with pharmaceutical industry players and agree on prices and essential medicines to be exempted was an enabler of policy implementation. Also, the institutional capacity of the Ghana Revenue Authority (GRA) to host the Integrated Customs Management Systems (ICUMS) portal for ease of application is an enabler for VAT exemption implementation.

 We also found three main contextual constraints to implementation of VAT exemption and FC policies. First, economic factors such as the depreciation of the local currency, increasing inflation rate, high bank interest rate, and increasing utility cost negatively affected the vendor’s ability to fulfill their orders under the FC policy and maintain lower prices for VAT exempted finished products. For example, the Bank of Ghana, Inter-Bank exchange rate (GH₵/US$) increased from 4.9506 in January 2019, to 5.4672 in January 2020, to 5.7604 in 2021, and 6.0236 in January 2022. (https://www.bog.gov.gh/economic-data/exchange-rate/).

 “*Economic factors such as bank interest rate, inflation and depreciating Ghana cedi are variables that negatively influence pharmaceutical prices and affect already agreed prices”* [Industry Association].

 The second constrain was the limited administrative capacities of (*a*) health facilities to pay debts and agreed prices on time and (*b*) vendors to quote competitive prices during the tendering processes.

 “*The challenge of indebtedness of facilities to suppliers is serious and they used this as a reason to not supply essentials medicines causing shortages”* [Public Sector].

 During the tendering processes, some vendors quoted unrealistic prices, which subsequently proved a challenge because they were unable to supply medicines at that price under the contract.

 “*The MoH must ensure that the prices quoted are competitive enough such that suppliers will not later say that they cannot supply because the contract prices are too low”* [Service Provider].

 Last, the impact of the COVID-19 pandemic. The pharmaceutical industry was not spared the brunt of COVID-19-related global supply chain disruption and shortage with increased shipping costs and delays. As noted by an importer during an interview, COVID-19 changed the supply chain cycle, importation timing and costs of finished products, active ingredients, and packaging materials became unpredictable, and these negatively affected the supply of medicines on time and at the agreed prices.

###  Interplay Between Policy Contents, Actors, and Contexts in Achieving Policy Outcomes 

 The interplay of policy content (nature and complexity), policy actors (participation and efficiency), and contextual feasibility (enablers and constraints) shaped the achievement of policy outcomes. In the implementation of the ‘at least’ 30% price reduction in 2018, data from documents reviewed and interviews show that expert TWG members initially decided to apply a 30% price reduction to the NHIS medicines list because the government is the largest buyer, and the pharmaceutical market is highly uncontrolled in terms of prices.^31^ However, not all NHIS medicines prices were reduced by 30%, during the process, and this appears to reflect the interrelatedness of policy content, actors’ actions, and context. The Ghana National Chamber of Pharmacy representatives argued that some medicine prices had been reduced since the passage and implementation of the VAT Regulations, (L.I) 2218 in 2015, and also price reductions will have to depend on medicines brand type and foreign exchange rate as economic context had bearings on how medicines were to be priced. Therefore, when the 30% reduction was applied to the NHIS’ 600 medicines, prices of 77 medicines were considered outliers. Prices of 11 out of the 77 medicines were further reduced and these included glimepiride 1mg tablet which was reduced by 50% from GH₵ 0.80 (2016 price) to GH₵ 0.40 (2018 price). The remaining 66 had higher prices than expected, for example, omeprazole 20mg tablet price was increased by 3%.^31^

 The interrelatedness of policy content, actors, and context on the implementation process was also evident during FC implementation. With unfavourable economic indicators affecting vendors’ ability to supply medicines at the agreed prices, vendors requested upward adjustments of the contract prices. The request was made to the MoH and approval was sought from the Ministry of Finance (MoF). Although there were no standardized price adjustment formulae for the FC implementation, the MoH managed to negotiate upward adjustments with the vendors. As noted by respondents, there were 8% and 20% upward price adjustments for phases 1 (2018) and 2 (2019) respectively and there is an ongoing discussion for an upward price adjustment for phase 3 (2020-2021).

 The interrelatedness of policy content, actors, and context influencing implementation outcomes give rise to five identified attributes of medicines pricing policy implementation: (1) policy nature and complexity, (2) inclusiveness, (3) organizational feasibility, (4) economic feasibility, and (5) political will and leadership. The descriptions are summarized in Table 3.

**Table 3:** Attributes of Medicines Pricing Policy Implementation

**Attributes **	**Description **
Policy nature and complexity	Clear and straightforwardness of policy aims and expected outcomes. Embeddedness in existing national policies
Inclusiveness	Active participation and effective management of stakeholders to ensure expected outcomes
Organizational feasibility	Organizational structures and technical experts to support the process
Economic feasibility	Economic drivers intended and unintended influences on the policy and how these are mitigated
Political will and leadership	Strong political support at all levels to lead and coordinate the process

## Discussion

 We analysed implementation of VAT exemptions and FC for essential medicines in Ghana, including influences over implementation from policy contents, actors and context.

 The use of FC and VAT exemptions to regulate medicines prices and promote availability and affordability is not unique to Ghana.^44,45^ However, implementation approaches vary as implementation structures and formalities, modalities, processes, and actors’ involvement are usually context-specific. Implementation of the VAT exemptions and FC policies in Ghana comprise a blend of ‘top-down’ and ‘bottom-up’ approaches to implementation. For both policies, decisions were mainly taken at the national level largely through legislative and administrative processes, reflecting ‘top-down’ policy-making.^46,47^ However, a ‘bottom-up’ approach, which focuses on negotiations, consensus building, and local discretion over implementation^46,47^ has also featured. Vendors, after signing contracts, withheld supplies from highly indebted facilities and renegotiated for upward price adjustments, reflecting their power to shape local implementation as ‘street level bureaucrats.’^48^

 Our results show that the nature and level of complexity of policy content shaped the implementation modalities such as the number of state implementing agencies. The broad nature of VAT exemption policy required involvement of multiple non-health state agencies, whereas implementation of a more technical framework agreement was mainly centered within the health sector. Policy clarity in terms of ensuring easy-to-understand language, clear roles and interactions among policy actors is critical for effective policy implementation.^47,49^ Our results also highlight the importance of having clear policy aims and expected outcomes aligned with supporting national laws and guidelines. While the former is well-recognized, the latter ie, consistency with other laws and guidelines is often implicit but is arguably critical for successful policy implementation.

 Our results illustrate the importance of participatory policy implementation, which can enable engagements of policy actors across multiple sectors for example through TWGs. Participatory approaches are critical and for designing and implementing policies,^50^ and can further augment the knowledge base of the policy processes^51^ for example through expert committees constituted for evidence sharing.^16,52^ In our study, the MoH actively engaged other state agencies and pharmaceutical industry throughout the implementation. The value of involvement of different stakeholders — such as pharmaceutical wholesalers, manufacturers, retailers, government agencies, health facilities and professional bodies — is also documented in other medicines pricing studies.^10,17,18,20^ There are claims that multi-stakeholder involvement contributes towards universal health coverage^53^ national health insurance system^54^ and research priority-setting.^55^

 In the case of the two examined policies, the implementation processes were however dominated by government agencies and pharmaceutical industry. Although the actors’ roles, powers, and resultant influences on the policy implementation are discussed elsewhere,^16^ personal commitments of some health facility managers and the Minister of Health are worth mentioning. Such policy champions are often critical to spearhead, shape and support policy initiatives,^56^ though of course a more balanced engagement of different stakeholders is important for shared ownership and effective implementation.

 Different contextual barriers and enablers shaped implementation of the two medicines pricing policies in Ghana. The main enablers included political support, multistakeholder engagement, and existing institutional structures and other laws augmenting VAT exemption and FC. As noted in other works, political support,^57^ engagement of different policy actors^58^ and the use of existing structures^59^ are important determinants of effective policy implementation. The contextual enablers can create different opportunities for policy actors,^60^ for example to act and work towards improving access to medicines. The main contextual barrier to policy implementation from our study was unfavourable economic environment comprising increasing inflation rate and depreciating local currency, echoing similar findings from South Africa.^61^

 Our results reinforce the argument that the interplay between policy context, processes, content, and actors can shape the achievement of policy outcomes.^38^ The policy outcomes in this study, which included price reduction and improved access, were mixed. For example, currency depreciation, disrupted supply chain due to COVID-19, and high indebtedness of some health facilities adversely affected the ability of vendors to supply medicines under the framework agreement. Furthermore, due to the influence from the powerful Ghana National Chamber of Pharmacy the 30% price reduction was not fully realized for all NHIS-listed medicines. On the other hand, existence of national procurement laws, VAT exemptions laws, and NHIS medicines list, all facilitated the timely implementation of the VAT exemptions that resulted in price reduction. As noted in an earlier study, contextual factors in an interrelating manner can serve as a constraint or an opportunity for timely and effective policy implementation.^62^

 The interrelatedness of policy content, actors, and context influencing implementation outcomes suggested five attributes of effective implementation of medicines pricing policies, shown in Table 3. These attributes are not specific to medicines pricing policies and thus can contribute to wider scholarship on health policy analysis through further advancing conceptualization of determinants of effective policy implementation.

 A recent systematic review of medicines pricing policies in sub-Saharan Africa revealed four domains of potential policy options: targeted public subsidies, regulatory frameworks and direct price control, generic medicine policies and purchasing policies.^5^ Ghanaian FC policy falls within the regulatory framework, whereas VAT exemptions policy mostly is an example of Government’s subsidy to reduce medicines pricing. While the focus of this study was on examining implementation of these two policies, the policy designers should also be aware of further policy options to reduce medicines pricing. It is equally important, however, to be cognizant of the context-specificity of different policy options with regard to the country’s policy, political, structural, and health systems context.

 Our analysis suggests four policy lessons and implications. First, having clearly defined policy objectives is equally important as having measures in place to mitigate effects of constraining contextual factors on policy implementation and outcomes. Second, a clear legitimacy and mandate to regulate medicines pricing is important, but there is also a need to balance the multiple agendas of different policy actors toward the expected policy outcomes. Third, appropriate sequencing of tasks to be performed by independent agencies and stakeholders during policy implementation is salient and demands attention, especially during participatory decision-making. Fourth, a clear and structured process can be a useful facilitator of effective policy implementation, but it needs to be simple enough for the policy actors to easily navigate the prescribed processes.

 We acknowledge two study limitations. First, we focused on VAT exemptions and FC only, and while we believe some broader lessons emerge from the study, some findings may not be generalizable to further medicines pricing policy options. Second, the implementation of these policies is ongoing, so the processes, structures, and contextual influences may further evolve. Despite these limitations, our analysis provides relevant and timely lessons to inform improvements to on-going implementation and facilitate achievement of policy outcomes.

## Conclusion

 Varied enabling and constraining contextual factors, role and active participation of stakeholders, nature of policy content, and structures influenced the implementation of VAT exemption and FC for selected essential medicines in Ghana. Understanding how and why context, policy content, stakeholders, and processes interact, and influence medicines pricing policy implementation outcome is therefore important in the design and implementation of interventions aimed at reducing medicines prices and improving access to medicines.

## Acknowledgements

 We acknowledge the NMPC members, MoH and our respondents.

## Ethical issues

 This study involves human participants. Ethical approval received from the ethics committees of the Ghana Health Service (GHS-ERC006/02/20) and the University of Leeds School of Medicine (MREC 19-060). Participants gave informed consent to participate in the study before taking part.

## Competing interests

 Authors are members of the NIHR-funded AMIPS (Access to Medicines through Improved Pricing Strategies) project a collaboration between the Universities of Ghana and Leeds, and the GHS, which sought to examine implementation of four medicine pricing policies in Ghana to inform improved policy implementation and contribute to improved access to essential medicines.

## Disclaimers

 Augustina Koduah is a member of the NMPC. The views expressed in this paper are solely the responsibilities of the named authors and do not necessarily reflect the decisions of the committee.

## Funding

 This research was commissioned by the National Institute for Health Research (NIHR) NIHR Global Health Policy and Systems Research Development Award using UK aid from the UK Government (grant number: 130219).

## Supplementary files

Supplementary file 1. Interview Guide.

Supplementary file 2. List of Essential Medicines for FC Phase 1.
