# Supplementary material for: Implementation of Medicines Pricing Policies in Ghana: The Interplay of Policy Content, Actors’ Participation, and Context
Source: Int J Health Policy Manag. 2023 Oct 11;12:7994. doi: 10.34172/ijhpm.2023.7994 (PMC10699811; doi:10.34172/ijhpm.2023.7994)
Supplement: Supplementary file 1 — Interview Guide. [file ijhpm-12-7994-s001.pdf]

Article title: Implementation of Medicines Pricing Policies in Ghana: The Interplay of Policy Content, Actors' Participation, and Context

Journal name: International Journal of Health Policy and Management (IJHPM)

Authors' information: Augustina Koduah[1]*, Leonard Baatiema[2], Irene A. Kretchy[1], Irene Akua Agyepong[3], Anthony DansoAppiah[4], Anna Cronin de Chavez[5], Timothy Ensor[6], Tolib Mirzoev[7]

[1]Department of Pharmacy Practice and Clinical Pharmacy, School of Pharmacy, University of Ghana, Legon, Ghana.

[2]Department of Health Policy, Planning & Management, School of Public Health, University of Ghana, Legon, Ghana.

[3]Public Health Faculty, Ghana College of Physicians and Surgeons, Accra, Ghana.

[4]Department of Epidemiology and Disease Control, School of Public Health, University of Ghana, Legon, Ghana.

[5]London School of Hygiene and Tropical Medicine, London, UK.

[6]Nuffield Centre for International Health, University of Leeds, Leeds, UK.

[7]Department of Global Health and Development, London School of Hygiene and Tropical Medicine, London, UK.

***Correspondence to:** Augustina Koduah; Email: akoduah@ug.edu.gh

Citation: Koduah A, Baatiema L, Kretchy IA, et al. Implementation of medicines pricing policies in Ghana: the interplay of policy content, actors' participation, and context. Int J Health Policy Manag. 2023;12:7994. doi:10.34172/ijhpm.2023.7994

Supplementary file 1. Interview Guide

Interview Guide

Participant information sheet and consent form will be sent to respondents and consent sought before conducting the interview.

The medicine pricing policies for our discussions are:

1. Framework contract for high demand medicines
2. Value Added Tax (VAT) exemptions for essential medicines and for active pharmaceutical inputs (API), manufacturing inputs and packaging materials

I. Introduction to respondents

1. Tell me about your organization and its areas of focus and role in the health sector?
2. Tell me about your role in the organisation and your specific areas of focus in relation to access to essential medicines?

II. Implementation approaches to policy implementation [for each medicine pricing policy]

1. Which implementation processes, mechanisms and timelines were anticipated, why and by whom? In your opinion were these implementation processes, mechanisms, and timelines appropriate and what would you recommend moving forward?
2. How are the policies currently implemented (in relation to implementation processes, steps, resources allocated, timing, involvement of actors, role of evidence etc) and why?
3. Which stakeholders were expected to engage in policy implementation, how and in what capacities or roles and why? How does this compare with any evidence of actual stakeholder involvements? What is your role in implementation process? Who else should be added and why? Were there stakeholders who opposed the implementation of these policies and why?
4. What other policies and laws facilitate or constrain this medicine pricing policy, how and why?

III. Evidence of policy implementation and facilitators and barriers [for each medicine pricing policy]

5. What (if any) is the evidence of effective implementation (or unsuccessful implementation) of these policies (in relation to reducing prices of medicines and improving access to services)?
6. What other policies and laws facilitate or constrain this medicine pricing policy, how and why?
7. Which other contextual facilitators affected the implementation of these policies, how and why? [For example, political, social, economic context etc.]
8. Which other contextual barriers affected the implementation of these policies, how and why?
9. Are there other health policies that are implemented well that we can learn from?
