# Supplementary material for: Implementation of Medicines Pricing Policies in Ghana: The Interplay of Policy Content, Actors’ Participation, and Context
Source: Int J Health Policy Manag. 2023 Oct 11;12:7994. doi: 10.34172/ijhpm.2023.7994 (PMC10699811; doi:10.34172/ijhpm.2023.7994)
Supplement: Supplementary file 2 — List of Essential Medicines for FC Phase 1. [file ijhpm-12-7994-s002.pdf]

Article title: Implementation of Medicines Pricing Policies in Ghana: The Interplay of Policy Content, Actors' Participation, and Context

Journal name: International Journal of Health Policy and Management (IJHPM)

Authors' information: Augustina Koduah[1]*, Leonard Baatiema[2], Irene A. Kretchy[1], Irene Akua Agyepong[3], Anthony DansoAppiah[4], Anna Cronin de Chavez[5], Timothy Ensor[6], Tolib Mirzoev[7]

[1]Department of Pharmacy Practice and Clinical Pharmacy, School of Pharmacy, University of Ghana, Legon, Ghana.

[2]Department of Health Policy, Planning & Management, School of Public Health, University of Ghana, Legon, Ghana.

[3]Public Health Faculty, Ghana College of Physicians and Surgeons, Accra, Ghana.

[4]Department of Epidemiology and Disease Control, School of Public Health, University of Ghana, Legon, Ghana.

[5]London School of Hygiene and Tropical Medicine, London, UK.

[6]Nuffield Centre for International Health, University of Leeds, Leeds, UK.

[7]Department of Global Health and Development, London School of Hygiene and Tropical Medicine, London, UK.

***Correspondence to:** Augustina Koduah; Email: akoduah@ug.edu.gh

Citation: Koduah A, Baatiema L, Kretchy IA, et al. Implementation of medicines pricing policies in Ghana: the interplay of policy content, actors' participation, and context. Int J Health Policy Manag. 2023;12:7994. doi:10.34172/ijhpm.2023.7994

Supplementary file 2. List of Essential Medicines for FC Phase 1

Lot No	Generic name, dosage form, strength	Unit size	Presentation
Lot 1	Albendazole Tablet 400mg	1 Tablet	Blister
Lot 2	Amitriptyline Tablet 25mg	1 Tablet	Blister
Lot 3	Amlodipine Tablet 10mg	1 Tablet	Blister
Lot 4	Amlodipine Tablet 5mg	1 Tablet	Blister
Lot 5	Amoxicillin+Clavulanic Acid Suspension 250mg+62mg, 70ml	1 Bottle	Bottle (Jacket)
Lot 6	Amoxicillin+Clavulanic Acid Suspension 400mg+57mg, 70ml	1 Bottle	Bottle (Jacket)
Lot 7	Amoxicillin+Clavulanic Acid Tablet 500mg+125mg	1 tablet	Blister
Lot 8	Amoxicillin+Clavulanic Acid Tablet 875mg+125mg	1 tablet	Blister
Lot 9	Amoxicillin Capsules 250mg	1 Capsule	Blister
Lot 10	Amoxicillin Capsules 500mg	1 Capsule	Blister

Lot No	Generic name, dosage form, strength	Unit size	Presentation
Lot 11	Artemether+Lumenfantrine Suspension (Powder for Reconstitution) 120mg/5ml	1 Bottle	Bottle (Jacket)
Lot 12	Bendroflumethiazide Tablet 2.5mg	1 tablet	Blister
Lot 13	Bendroflumethiazide Tablet 5mg	1 tablet	Blister
Lot 14	Carbocistiene Syrup 250mg/5ml, 100ml	1 Bottle	Bottle
Lot 15	Cefuroxime Suspension 125mg/5ml, 50ml	1 Bottle	Jacket
Lot 16	Cefuroxime Tablet 250mg	1 Tablet	Blister
Lot 17	Cetirizine Tablet 10mg	1 tablet	Blister
Lot 18	Ciprofloxacin Eye Drop 0.3%, 10ml	1 Bottle	Bottle
Lot 19	Ciprofloxacin Tablet 500mg	1 tablet	Blister
Lot 20	Clindamycin Capsules 150mg	1 capsule	Blister
Lot 21	Dexamethasone Eye Drops 1%, 5ml	1 Bottle	Bottle
Lot 22	Diclofenac Capsules 75mg	1 capsule	Blister
Lot 23	Diclofenac Gel, 30g	1 Tube	Tube
Lot 24	Diclofenac Injection 75mg/3ml	1 Ampoule	Ampoule
Lot 25	Diclofenac Suppository 100mg	1 suppository	Blister
Lot 26	Diclofenac Tablet 50mg	1 tablet	Blister
Lot 27	Ferrous Sulphate Tablet 60mg (Elemental Iron)	1 tablet	Blister/ Loose
Lot 28	Flucloxacillin Capsules 250mg	1 capsule	Blister
Lot 29	Folic Acid Tablet 5mg	1 tablet	Blister
Lot 30	Glibenclamide Tablet 5mg	1 tablet	Blister
Lot 31	Glimepiride Tablet 2mg	1 tablet	Blister
Lot 32	Glimepiride Tablet 4mg	1 tablet	Blister
Lot 33	Ibuprofen Tablet 400mg	1 tablet	Blister
Lot 34	Insulin Premixed (30/70) HM Injection 100 units/ml in 10ml	1 vial	Vial
Lot 35	Iron (III) Polymatose Complex Capsules	1 capsule	Blister
Lot 36	Iron (III) Polymatose Complex Suspension, 200ml	1 Bottle	Bottle
Lot 37	Lisinopril+Hydrochlorthiazide Tablet (10mg+12.5mg)	1 tablet	Blister
Lot 38	Lisinopril Tablet 10mg	1 tablet	Blister
Lot 39	Losartan Tablet 100mg	1 tablet	Blister
Lot 40	Losartan Tablet 50mg	1 tablet	Blister
Lot 41	Metformin Tablet 500mg	1 tablet	Blister
Lot 42	Methyldopa Tablet 250mg	1 tablet	Blister
Lot 43	Metronidazole Tablet 200mg	1 tablet	Blister
Lot 44	Metronidazole Tablet 400mg	1 tablet	Blister

Lot No	Generic name, dosage form, strength	Unit size	Presentation
Lot 45	Multivitamin Syrup, 125ml	1 Bottle	Bottle
Lot 46	Multivitamin Tablet	1 tablet	Blister
Lot 47	Nifedipine Tablet 20mg (slow release)	1 tablet	Blister
Lot 48	Nifedipine Tablet 30mg (GITS)	1 tablet	Blister
Lot 49	Omeprazole Capsule 20mg	1 Capsule	Blister
Lot 50	Oral Rehydration Salt Powder	1 Sachet	Sachet
Lot 51	Paracetamol Syrup 120mg/5ml, 125ml	1 Bottle	Bottle
Lot 52	Paracetamol Tablet 500mg	1 Tablet	Blister
Lot 53	Simple Linctus BPC (Paediatric), 125ml	1 Bottle	Bottle
Lot 54	Simple Linctus BPC, 200ml	1 Bottle	Bottle

List of essential medicines for FC phase 2 and 3

Lot No	Generic Name, Dosage Form, Strength	Unit Size	Presentation
Lot 1	Adrenaline injection, 1:10,000, 1ml	1 Vial	Vial
Lot 2	Albendazole Suspension 100mg/5ml in 20ml	1 Bottle	Bottle
Lot 3	Albendazole Tablet, 400 mg	1 Tablet	Blister
Lot 4	Amitriptyline Tablet, 25 mg	1 Tablet	Blister
Lot 5	Amlodipine Tablet, 10 mg	1 Tablet	Blister
Lot 6	Amlodipine Tablet, 5 mg	1 Tablet	Blister
Lot 7	Amoxicillin + Clavulanic Acid injection,1000mg+200mg	1 Vial	Vial
Lot 8	Amoxicillin + Clavulanic Acid Suspension, 200mg +28.5mg/5ml, 70ml	1 Bottle	Bottle
Lot 9	Amoxicillin + Clavulanic Acid Suspension, 400mg +57 mg/5ml, 70 ml	1 Bottle	Bottle
Lot 10	Amoxicillin + Clavulanic Acid Tablet, 500 mg + 125 mg	1 Tablet	Blister
Lot 11	Amoxicillin + Clavulanic Acid Tablet, 875 mg + 125 mg	1 Tablet	Blister
Lot 12	Amoxicillin Capsule, 250 mg	1 Capsule	Blister
Lot 13	Amoxicillin Capsule, 500 mg	1 Capsule	Blister
Lot 14	Anti Rabies Injection	1 Vial	Vial
Lot 15	Anti Snake serum, (polyvalent) 10ml	1 Vial	Vial
Lot 16	Anti Tetanus Serum Injection 1500 IU	1Ampoule	Ampoule
Lot 17	Atorvastatin Tablet, 10mg	1 Tablet	Blister
Lot 18	Atorvastatin Tablet, 20mg	1 Tablet	Blister
Lot 19	Bendroflumethiazide Tablet, 2.5 mg	1 Tablet	Blister
Lot 20	Carbocisteine Syrup, 250 mg/5 mL, 100ml	1 Bottle	Bottle
Lot 21	Cefuroxime Suspension, 125 mg/5 ml, 50ml	1 Bottle	Bottle
Lot 22	Cefuroxime Tablet, 250 mg	1 Tablet	Blister
Lot 23	Cetirizine Tablet, 10 mg	1 Tablet	Blister
Lot 24	Ciprofloxacin Eye Drops, 0.3%, 10ml	1 Bottle	Bottle
Lot 25	Ciprofloxacin Tablet, 500 mg	1 Tablet	Blister

Lot No	Generic Name, Dosage Form, Strength	Unit Size	Presentation
Lot 26	Clindamycin Capsule, 150 mg	1 Capsule	Blister
Lot 27	Dexamethasone Eye Drops, 1%, 10ml	1 Bottle	Bottle
Lot 28	Diazepam injection 5mg/ml, 2ml	1 Ampoule	Ampoule
Lot 29	Diclofenac Capsule, 75 mg	1 Capsule	Blister
Lot 30	Diclofenac Gel 30g	1 Tube	Tube
Lot 31	Diclofenac Injection, 75mg/3ml in 3ml	1 Ampoule	Ampoule
Lot 32	Diclofenac Suppository, 100 mg	1Suppository	Blister
Lot 33	Diclofenac Tablet, 50 mg	1 Tablet	Blister
Lot 34	Ferrous Sulphate Tablet, 60 mg (Elemental Iron)	1 Tablet	Blister
Lot 35	Flucloxacillin Capsule, 250 mg	1 Capsule	Blister
Lot 36	Folic Acid Tablet, 5 mg	1 Tablet	Blister
Lot 37	Furosemide injection, 10mg/ml in 2ml	1 Ampoule	Ampoule
Lot 38	Glimepiride Tablet, 2 mg	1 Tablet	Blister
Lot 39	Glimepiride Tablet, 4 mg	1 Tablet	Blister
Lot 40	Ibuprofen Tablet, 400 mg	1 Tablet	Blister
Lot 41	Insulin premixed (30/70) HM Injection, 100 units/mL in 10 mL	1 Vial	Vial
Lot 42	Insulin Soluble HM, Injection100 units/ml in 10ml	1Vial	Vial
Lot 43	Iron (III) Polymaltose Complex Capsule, 100mg Elemental Iron	1 Capsule	Blister
Lot 44	Iron (III) Polymaltose Complex Suspension, 50mg/5ml, 200ml	1 Bottle	Bottle
Lot 45	Lisinopril Tablet, 10 mg	1 Tablet	Blister
Lot 46	Losartan Tablet, 100 mg	1 Tablet	Blister
Lot 47	Losartan Tablet, 50 mg	1 Tablet	Blister
Lot 48	Metformin Tablet, 500 mg	1 Tablet	Blister
Lot 49	Methyldopa Tablet, 250 mg	1 Tablet	Blister
Lot 50	Metronidazole Tablet, 200 mg	1 Tablet	Blister
Lot 51	Metronidazole Tablet, 400 mg	1 Tablet	Blister
Lot 52	Morphine Injection 10mg/ml in 1ml	1 Ampoule	Ampoule
Lot 53	Multivitamin Syrup 125ml	1 Bottle	Bottle
Lot 54	Multivitamin Tablet	1 Tablet	Blister
Lot 55	Nifedipine Tablet, 20 mg (slow release)	1 Tablet	Blister
Lot 56	Nifedipine Tablet, 30 mg (GITS)	1 Tablet	Blister
Lot 57	Omeprazole Capsule, 20 mg	1 Capsule	Blister
Lot 58	Oral Rehydration Salts Powder	1 Sachet	Sachet
Lot 59	Oxytocin injection 10 iu/ml in 1ml	1 Ampoule	Ampoule
Lot 60	Paracetamol Syrup, 120 mg/5 mL, 125ml	1 Bottle	Bottle
Lot 61	Paracetamol Tablet, 500 mg	1 Tablet	Blister
Lot 62	Phytomenadione (Vitamin K1) injection 1mg/ml paediatric (water soluble) IM	1 Ampoule	Ampoule
Lot 63	Prednisolone Tablet, 5mg	1 Tablet	Blister
Lot 64	Simple Linctus BPC (Peadiatric) Citric Acid Monohydrate 31.25mg/5ml, 100ml	1 Bottle	Bottle
Lot 65	Simple Linctus BPC (Adult) Citric Acid Monohydrate 125mg/5ml,100ml	1 Bottle	Bottle
